# Barriers and facilitators of antiseizure medication adherence: a qualitative study among persons with epilepsy in Pakistan

**DOI:** 10.1186/s12883-025-04433-9

**Published:** 2025-10-14

**Authors:** Bushra Batool Zahra, Muhammad Amir Hamza, Rehana Sarwat, Sharon Floric, Maria Tanveer, Anam Fatima, Muhammad Tariq Khan, Mudassar Iqbal Arain, Ali Ahmed

**Affiliations:** 1 Department of Pharmacy, Faculty of Pharmaceutical Sciences, Riphah International University, Islamabad, Pakistan; 2 Department of Neurology, Pakistan Institute of Medical Science (PIMS), Islamabad, Pakistan; 3 School of Clinical Sciences, Faculty of Health, Queensland University of Technology, Brisbane, QLD Australia; 4 Faculty of Pharmacy, The University of Lahore, Lahore, Pakistan; 5 Department of Pharmacy, Faculty of Biological Sciences, Quaid-I- Azam University, Islamabad, Pakistan; 6 Clinical Research Program, College of Health and Human Services, University of North Carolina, Wilmington, NC USA; 7 Division of Infectious Diseases and Global Public Health, School of Medicine, University of California San Diego (UCSD), La Jolla, San Diego, CA USA

**Keywords:** Chronic neurological disease, Experiences, Drug resistance, Health hazards, Comorbidities, Patient safety

## Abstract

**Background:**

Medication adherence is crucial to achieving optimal health outcomes in persons with epilepsy. Approximately 52.1% of persons with epilepsy are sub-optimally adherent to antiseizure medications (ASMs) in Pakistan, leading to increased risk of drug resistance and mortality. Achieving adherence in Pakistan is challenging because of socioeconomic, psychological, and cultural factors. In this qualitative study, we identified the factors that hinder or support adherence to medication regimens and explored the lived experiences of persons with epilepsy regarding adherence to ASMs.

**Methods:**

Twenty-eight qualitative interviews were conducted with persons with epilepsy who visited the largest hospital in Islamabad, Pakistan Institute of Medical Sciences (PIMS), from March to June 2024. Clinically diagnosed persons with epilepsy with their caregivers (CGs), were invited to participate in this study. This study employed a phenomenological study design and purposive sampling method to achieve data saturation. Interviews averaged 30 min in length and were conducted using a semi-structured guide. Interview questions were open-ended and designed to elicit barriers and facilitators to ASMs adherence among persons with epilepsy and their CGs. All interviews were audio-recorded in Urdu and transcribed into English. The qualitative responses were analyzed using a thematic analysis technique emphasizing the dominant themes.

**Results:**

Three themes emerged surrounding issues of persons with epilepsy and their CGs in adhering to ASMs, encompassing factors related to the persons with epilepsy, healthcare, and medications. Persons with epilepsy related factors includes stigma, psychological issues, financial instability, comorbidities, and family support. Healthcare-related factors include short consultation times, free-of-cost treatment facilities, and availability of healthcare providers. Medication-related factors include non-availability of ASMs, side effects, and perceived treatment efficacy.

**Conclusions:**

The findings of this study can be valuable for policymakers in formulating policies for epilepsy care and treatment. Enhancing the reported facilitators, such, providing free-of-cost treatment facilities, and the availability of ASMs in poor-resourced areas, will improve health outcomes and ensure the continuum of epilepsy care among persons with epilepsy residing in Pakistan.

**Supplementary Information:**

The online version contains supplementary material available at 10.1186/s12883-025-04433-9.

## Background

Epilepsy is a common, chronic, treatable neurological disorder characterized by recurrent seizures, which affects almost 70 million people worldwide [1]. Epilepsy is a stigmatized neurological disease in low- and middle-income countries (LMICs), and its prevalence is higher in Asian countries due to a lack of resources to procure treatment [2]. Active epilepsy prevalence in developing countries is higher than in developed countries and ranges from 0.5 to 1.0% [3]. In Pakistan, the prevalence of epilepsy is estimated to be 9.99/1000; however, recent epidemiological data are unavailable [4]. In LMICs, epilepsy often leads to an increase in physical and mental disability due to delayed or inappropriate treatment [5].

Antiseizure medications (ASMs) are the Mainstay of effective treatment, controlling recurrent seizures and ultimately improving the quality of life. Previous data suggest that approximately 70⁒ of persons with epilepsy achieve seizure remission with consistent adherence to appropriate ASMs [6]. In LMICs, achieving optimal medication adherence is challenged by a range of psychosocial, cultural, and economic factors [7]. Successful Epilepsy Management and a seizure-free life depend on consistent adherence to treatment, including medication and self-care practices. However, in LMICs, approximately 75⁒ of persons with epilepsy either do not receive treatment or fail to maintain adherence [8]. Medication nonadherence can result in serious consequences, including increased risk of injury and accidents, depression, reduced autonomy and mobility, extended hospitalizations, and ultimately, unsuccessful epilepsy control due to progressive neuronal damage [9].

Numerous studies have identified both barriers and facilitators in ASMs adherence among persons with epilepsy, including negative beliefs about medications, forgetfulness, poor knowledge, and unhelpful attitudes [10–13]. Conversely, facilitators such as self-care practices, adequate treatment knowledge, and strong family support have been shown to promote adherence [14, 15]. However, no qualitative studies to date have explored these factors in the context of Pakistan. As a developing country with ongoing challenges in health care infrastructure, Pakistan presents a unique cultural landscape [16]; low health literacy, diverse ethnic backgrounds, strong traditional beliefs, and a heavy reliance on caregivers (CGs) characterize its population. These factors, combined with limited health sector capacity, suggest that findings from international studies may not be directly applicable in this setting [17]. Therefore, we conducted an in-depth qualitative study to capture lived experiences and internal patients’ experiences surrounding ASMs adherence faced by persons with epilepsy and their CGs in Pakistan. The knowledge, attitudes, and practices of CGs play a critical role in epilepsy care, as they significantly influence the help-seeking behavior of persons with epilepsy [18]. This study aimed to explore the barriers and facilitators to medication adherence to identify actionable factors that could enhance adherence to ASMs.

## Methods

### Study setting and design

This qualitative research followed the Consolidated Criteria for Reporting on Qualitative Research (COREQ) [19] (Supplementary File 1). A phenomenological study design was employed to comprehend the lived experiences of persons with epilepsy [20]. The study was conducted at the Pakistan Institute of Medical Sciences (PIMS) neurology department, Islamabad. The institute has a specialized 24-bed care unit for persons with epilepsy, equipped with the latest neurodiagnostic facilities [21].

### Participant sampling and recruitment

A total of twenty-eight persons with epilepsy were recruited through purposive sampling to gather diverse data in age, gender, education level and urban/rural residence at PIMS by the Principal Investigator, B.B.Z, alongside S.F., a hospital pharmacist involved in the study. The demographic data only included the socio-demographic characteristics of the participants. Participants were divided into two key informant groups: persons with epilepsy themselves and persons with epilepsy dependent on their CGs. Eligibility criteria for the study included Individuals aged 18 years and above, capable of giving written informed consent, and clinically diagnosed with epilepsy. For persons with epilepsy who could not provide their medication information, interviews were conducted with their CGs, who assisted in supplying the necessary data. Exclusion criteria included persons with epilepsy with insufficient verbal communication skills, individuals over 65 years, and those with a medication history of less than three months.

### Interview guide development and data collection procedure

A preliminary semi-structured interview guide based on an extensive literature review [9, 22–27] was developed after discussion with neurologists at the PIMS neurology department. Interview questions that aligned with the current study’s objectives were included. The guide was validated using argumentative and cumulative methods, enhancing its credibility and ensuring reliability for consistent data collection [28]. Three pilot interviews from participants were conducted to ensure that the interview guide covers all causes of sub-optimal medication adherence.The data from three pilot interviews were not included in the final Thematic Content Analysis (TCA) [29]. The semi-structured interview guide used in this study is available in Supplementary File 3.

B.B.Z. conducted the interviews. The interviews were audio recorded using a digital audio recorder, and participants were allowed to provide information during their follow-up and admission to PIMS. Interviews were recorded from March to June 2024, and each interview lasted 25 to 30 min. To ensure privacy, recorded interviews were assigned pseudonyms for de-identification and anonymity. B.B.Z. has securely stored all interview recordings and transcript files in an online repository (Google Drive). B.B.Z. substituted participant identifiers with pseudonyms promptly after transcription. Each transcript assigned was designated with a unique alphanumeric code (e.g., P 01). The numbers represent the sequence in which the participants were recruited. All the identifiable data were anonymized and encrypted only accessed by B.B.Z. and the senior author A.A. Interviews were conducted in the local Urdu language to ensure comfort and understandability among participants. The rationale for selecting twenty-eight participants was determined by data saturation [30], which was reached when interviews demonstrated no new themes or additional issues. Response saturation occurred at the 25th interview, and a further three interviews were conducted to validate the saturation of responses in interviews [31].

### Data analysis

An inductive analysis was used to examine the transcript to identify the themes and make subthemes from them [32]. Audio-recorded interviews were first downloaded on a laptop and transcribed in English. After transcribing, coding was done manually to highlighted lines of text in the transcripts using the emergent thematic framework approach [33]. Throughout the transcription process, potential codes were identified, subsequently integrating them with final codes to produce a comprehensive list of significant themes and sub themes for the study. Themes from each interview transcript were meticulously documented, evaluated, systematically compared, and refined to precisely select and identify the significant codes that effectively represented the participants’ perspectives. B.B.Z. finalized the initial coding, which was later assessed for the identification of emerging themes and subthemes by Co-authors (M.A.H, R.S.). Redundant codes were removed, and similar codes were systematically organized and categorized according to their relevant themes. Once the coding system achieved consistency, it underwent a comprehensive review by the entire study team, supplemented by biweekly Zoom meetings with the A.A. to ensure the data reliability and accuracy of the results’ interpretation. Furthermore, thematic development was informed by consultation with published literature [34–36]. Direct participants quotations are italized, coded (e.g., P12 caregiver/male/female), and serve as representative excerpts.

### Ethical considerations

The ethical review committee at Shaheed Zulfiqar Ali Bhutto University, Islamabad, approved the research study with reference number F.1–1/2015/ERB/SZABMU/1251. The study was conducted in adherence with the guidelines outlined in the Declaration of Helsinki [37]. Informed written consent forms were obtained for each participant. For participants with limited literacy, B.B.Z. verbally informed them about the recorded interview, the purpose and its benefits. Researchers maintained reflexivity through journaling and team discussions to limit personal biases influencing interpretation.

## Results

### Demographic characteristics of participants

The sample was comprised of participants with a mean age of 42 years, comprising (*n* = 18) males, (*n* = 10) females, having no primary education (*n* = 15), residing in rural areas (*n* = 15), and living a married life (*n* = 13). The details of participant characteristics are presented in Table 1. Our analysis yielded three main themes: (1) Persons with epilepsy-related factors, (2) Healthcare -related factors, and (3) Medication-related factors. These themes were further submerged into sub-themes. We provide key quotes that illustrate and validate the primary themes in the results section, with further quotes accessible in Supplementary File 2.

**Table 1:** Demographic characteristics of study participants

Characteristics	Category	Persons with epilepsy *n* (%)	Care givers *n* (%)	Total *n* (%)
Gender	Male	12(42%)	6(21%)	18(64%)
	Female	2(7%)	8(28%)	10(36%)
Age	< 18	8(28%)	9(32%)	16(57%)
	20–40	6(21%)	4(14%)	8(29%)
	41–60	1(3%)	3(11%)	4(14%)
	> 60	0(0%)	1(3%)	1(3%)
Marital status	Single	9(32%)	3(11%)	12(49%)
	Married	3(11%)	10(36%)	13(46%)
	Divorced	2(7%)	1(3%)	3(11%)
Education	Un educated	10(36%)	5(18%)	15(54%)
	Primary school	3(11%)	6(21%)	9(32%)
	Higher education	1(3%)	3(11%)	4(14%)
Residence	Urban	11(39%)	9(32%)	13(46%)
	Rural	3(11%)	5(18%)	15(54%)

### Persons with Epilepsy- related factors

Persons with epilepsy-related factors describe participants experiences, which includes their behavior, attitude, and religious beliefs in the management of their illness.

### Disease related stigma and discrimination

Due to disease stigma, participants reported negative behavior, which leads to social isolation and reduced access to healthcare.

*I become uncomfortable; I do not like to talk with anyone*,* and eventually*,* a headache starts. I want to fight with people and become aggressive -P4*,* 26 years*,* male.*

Participants reported revealing their medical condition in front of any person other than their family will lead to their maladjustment in society.

*She usually does not prefer to go outside the home; she only visits her grandmother’s house and easily takes her medicine for seizures there -P6*,* 52 years*,* female caregiver.*

### Psychological issues and comorbidities

Participants reported significant psychological distress and perception of reduced personal value, with a few participants reporting feeling unheard. Psychological screening and mental health support can prevent psychologically ill persons with epilepsy from harmful consequences.

*When I am stressed after an attack*,* I prefer to sleep and not take medicine for seizures -P26*,* 31 years*,* female.*

A few participants said that comorbidities hinder their regular illness management practice, especially ASMs consumption.

*He cannot take medicines because excessive saliva is coming from his mouth*,* and he is bedridden; we cannot bring him to the hospital for regular checkups. -P20*,* 50 years*,* male caregiver.*

### Financial instability

Mostly, interviewed participants belong to rural areas, having lower middle-class backgrounds, suffering from financial crises. They cannot afford treatment in private hospitals due to the expensive epilepsy treatment facilities and transportation cost.

*It is difficult to manage transport costs*,* but we have no other option. We can only provide our son with treatment*,* but not quality treatment. - P21*,* 30 years*,* female caregiver*.

### Religious practices

Participants reported that they usually skipped their daytime dose to meet their religious obligations, such as fasting during Ramadan.

*In Ramadan*,* I missed my medicine for seizures morning dose due to fasting. -P10*,* 41 years*,* female.*

### Fear of epileptic attack pain

Participants reported experiencing significant fear regarding epileptic seizures, as these episodes could occur unexpectedly, potentially leading to falls in public and involuntary body movements. Following the attack, the discomfort in their bodies becomes intolerable. To alleviate this discomfort, they utilized their ASMs appropriately.

*Now*,* I’ve become fearful about the pain from epileptic attacks. My hands and feet start bending*,* and unbearable pain starts. If I do not have money to buy medicine for seizures*,* I buy one tablet from the pharmacy and take it*,* but I never miss it- P01*,* 28 years*,* female*.

### Family support and sense of responsibility

Some of the interviewees reported that they did not know about their disease, as their family never told them. They gradually know due to the follow-ups in neurology care centers and their CGs conversations with neurologists.

*Yes*,* I have my family back*,* so I have survived despite this horrible disease. My sister gave me medicine for seizures properly with full attention. Now I’m recovering - P12*,* 20 years*,* female*.

Interviewees said that they are the only breadwinners in their families and want to live a epilepsy-free life for them. A sense of responsibility was the reason that kept them adhering to ASMs, and they did not miss ASMs dose.

*My family is dependent on me*,* and my children are growing. I want to live an epilepsy-free life for them. - P16*,* 44 years*,* male*.

#### Healthcare-Related factors

Four subthemes have emerged, intricating ASMs adherence challenges.

### Short consultation times

Participants expressed that due to long duty hours and high census of patients, the healthcare staff attending them cannot focus on single person with epilepsy.

*We have only access to the healthcare staff counter*,* The doctor is occupied with too many patients. Usually*,* the staff comes and injects the medicines and only informs us about the medicine for seizures -P23*,* 24 years*,* male caregiver*.

Many interviewees reported that they were referred to PIMS due to the wrong diagnosis by physicians working in their hometown.

*My doctor in my hometown in Kashmir stopped my medicine for seizures after my first epileptic attack by diagnosing me with facial paralysis. - P4*,* 21 years*,* female*.

The participant expressed that the neurologist did not thoroughly consider their condition. The prolonged queue of persons with epilepsy waiting outside results in brief interactions, where they primarily observe and prescribe medications during follow-up appointments.

*The doctor only sees the previous prescription and writes new medicines*,* and my turn ends in five minutes - P9*,* 30 years*,* female*.

### Neurology residents’ limited knowledge

Participants said that prescribing decisions were often made after residents consulted mobile applications or online resources, indicating a lack of confidence in independent clinical decision-making.

*These doctors in training usually prescribe me the medicines after confirming them through mobile phones. - P11*,* 35 years*,* male*.

### Free of cost epilepsy treatment facilities

The provision of free-of-cost treatment including medications in PIMS was a significant facilitator, enabling participants to maintain adherence to ASMs and overall treatment.

*Due to my financial issues*,* free-of-cost medicine for seizures are available here*,* the reason of my epilepsy free life for the last five years - P3*,* 36 years*,* female*.

As reported by participants, the hospitals and clinics in their villages lack adequate treatment facilities for treatment. Upon visiting PIMS, individuals feel relieved as they have access to free treatment facilities.

*My son was referred to PIMS because they have the only facilities and medication to treat epilepsy without any charges -P19*,* 56 years*,* male caregiver*.

### Healthcare providers availability

A highly appreciated facilitator reported by participants was the availability of a healthcare provider 24/7 in PIMS.

*Whenever I came to the hospital during an emergency condition*,* doctors were available and they tackled my miserable condition immediately due to seizures - P7*,* 24 years*,* male*.

Participants expressed a heightened sense of security while disclosing their experiences and illnesses to their neurologist, as they had trust and confidence in their treatment capabilities.

*Firstly*,* I confirm the medicine from the doctor*,* then I take the medicine without his consent I don’t take any medicines unless he prescribes it - P18*,* 51 years*,* male*.

#### Medication-Related factors

The following subthemes describe participants experiences regarding their prescribed ASMs.

### Non-availability of ASMs

ASMs non-availability presented significant obstacles for participants. Prolonged delays in ASMs availability and availability of alternate brands of prescribed ASMs in hospital pharmacies results in dissatisfaction for consumption of ASMs.

*No*,* I did not purchase the alternate brand of prescribed medicines. Rather*,* I prefer to wait and omit my dose until the prescribed brand of medicine is not available in the market -P27*,* 26 years*,* male.*

### Medication side effects and pill burden

Participants reported that due to the multiple medications burden, they cannot consume all prescribed ASMs daily as they suffer from side effects such as stomach upset, lethargy, and drowsiness.

*Due to his multiple medicines for cerebral palsy*,* he usually skips some medicine for seizures doses due to upset stomach. He cannot take all the medicines as he feels full the time*,* and we have to maintain six-hour medicines gap - P25*,* 33 years*,* male caregiver.*

Participants reported they were taking half of the prescribed ASMs doses without confirming it with their prescriber. This challenge reflects a lack of medication counseling, and suboptimal ASMs consumption leads to nonadherence.

*I usually skip my morning medicine for seizures dose and only take it at night*,* as it is easy for me because in the morning it causes drowsiness–P19*,* 31years*,* male*.

### Self-adjustment and perceived efficacy

A notable issue highlighted was the authority of persons with epilepsy to make decisions regarding their prescribed ASMs, which included improper administration, omission of doses, or switching to an unapproved ASM not sanctioned by their healthcare providers. This challenge reflects poorly monitored medication treatment.

*I usually double my medicine for seizures dose when I suffer from seizures during my sleep time. I feel very sleepy when I wake*,* and due to this fatigue*,* I forget to take my medicines at regular times –P21*,* 26 years*,* female*.

### Perceived treatment efficacy and torelability

Many respondents said they are punctual in their follow-up and medication routine due to effective monotherapy like levetiracetam.

*Yes*,* I use medicines from multiple companies*,* but the tablet LERACE is very effective for me. When I was using other medicine for seizures before Lerace*,* I still suffered from fits*,* but after taking Lerace*,* I am seizure-free - P1*,* 31 years*,* female.*

Participants reported feeling better after taking ASMs such as valproic acid in the morning because they act as mood stabilizers. These considerations keep them adhering to the ASMs.

*I feel very normal after taking my morning medicine for seizures. I feel calm and relaxed*,* and my mood gets better. -P10*,* 36 years*,* male*.

## Discussion

This study explored the experiences of key informants regarding adherence to ASMs in primary healthcare settings in Pakistan. Acknowledging the established beneficial role of ASMs adherence, our findings highlighted the difficulties faced by a diverse group of persons with epilepsy in practising medication adherence [38–41]. The participants in this study expressed that stigma, medication side effects, and the non-availability of ASMs lead to poor implementation of the correct treatment protocol. Additionally, they highlighted facilitators of medication adherence, such as family support, availability of neurologists, and free-of-cost treatment, as the primary enablers.

Multiple medications’ side effects and short consultation times were alarming barriers reported by participants suffering from comorbidities. Previous studies have shown these factors contribute to side effects, diagnosis delays, and higher treatment costs [42–44]. In developed countries, studies show pharmacist-led educational interventions have revolutionized epilepsy treatment with better treatment outcomes [45]. The inclusive approach of pharmacists in the multidisciplinary care process in the neurology department is highly needed, as they can significantly reduce the barriers to medication adherence. They will not only assist persons with epilepsy with difficulty regarding any prescribed ASMs but also reduce the burden on healthcare providers, side effects, and communication gaps [46–50].

Stigma and discrimination were significant barriers among many respondents. Previous research papers reported that a lack of awareness among the local public is the primary reason for increased stigma and discrimination among persons with epilepsy. Different awareness campaigns have been led to improve the quality of life of persons with epilepsy [51, 52]. Such initiatives involving treatment awareness campaigns and programs through social media in Pakistan can reduce the epilepsy burden.

Beyond stigma and discrimination, primary reported barriers to adherence, many historical factors indicate that epilepsy has been affected by the religious experiences and longstanding cultural beliefs of persons with epilepsy [53]. According to studies, 96.4% of the population in Pakistan follows Islam. In Ramadan, the medication regimen needs alteration [28]. There is a need for culturally tailored counseling, and religious scholars should work with healthcare professionals to guide persons with epilepsy accordingly.

The study highlighted the non-availability of ASMs as a significant barrier to ASMs adherence. Previous research indicates that almost 80% of the population in LMICs faces a non-availability of ASMs and lacks reliable access to free-of-cost treatment [54, 55]. We advocate for provincial programs to subsidize ASMs in rural pharmacies and government hospitals should be well-equipped and provide free of cost treatment to financially unstable persons with epilepsy because a large population of persons with epilepsy visits hospitals and clinics offering free treatment in countries with higher poverty rates [56]. Participants experienced long hospital admissions due to poor diagnosis. Natasha Alessi et al. reported that poor diagnosis of epilepsy can cause poor epilepsy treatment [57]. Government hospitals should initiate diagnostic training to prevent the underprivileged high-risk population.

Family support, including the support from CGs, was an effective ASMs adherence factor. A systematic review indicated the significance of providing families with detailed epilepsy information for the well-being of persons with epilepsy [58]. The research has reported that CGs burden is a recognized factor contributing to persons with epilepsy diminished quality of life [59]. Consequently, our study indicates the practical significance of acknowledging CGs as partners in medication adherence, offering training and education to enhance medication treatment knowledge, ultimately improving the healthcare outcomes in persons with epilepsy.

Future qualitative studies on ASMs adherence should systematically capture epilepsy type, number of ASMs, and illness duration along with sociodemographic characteristics, and evaluate the impact of pharmacist-led educational intervention among persons with epilepsy, and report the barriers to implementing the prescribing services for persons with epilepsy. Different considerations for policymakers and countermeasures have been proposed in Table 2 to remove the obstacles to ASMs adherence.

**Table 2:** Summary of considerations for policymakers and countermeasures to address ASMs^a^ adherence challenges among persons with epilepsy and their caregivers

Themes	Subthemes	Countermeasures (Practical Action)	Policy Recommendations (System Level)
Persons with Epilepsy- Related Factors	Stigma & Discrimination	Launch epilepsy awareness campaigns via schools, workplaces, and the media	National epilepsy awareness programs endorsed by the Ministry of Health to reduce stigma and encourage disclosure
	Psychological issues & Comorbidities	Provide screening and referral pathways for depression and anxiety Inclusion of empathy (Socio-psychological support)	Integrate mental health support services into neurology clinics for holistic care. Inclusion of empathy and compassion training in the medical curriculum.
	Financial instability	Introduce hospital-based social welfare desks and transport subsidies	Government-financed patient support programs for low-income families (e.g., Bait-ul-Mal)
	Religious practices (Ramadan fasting, spiritual healing)	Develop culturally tailored counseling with input from religious scholars	Policy-level integration of religious leaders into health promotion campaigns on ASM adherence
	Family support & Sense of responsibility	Provide caregiver education programs	National caregiver training initiatives for epilepsy care
Healthcare-Related Factors	Short consultation times	Delegate adherence counseling tasks to clinical pharmacists and trained nurses	Policy to incorporate pharmacists into epilepsy multidisciplinary teams
	Neurology residents’ limited knowledge	Structured resident training on epilepsy treatment	Curriculum reform: include ASM^a^ prescribing & counseling in residency programs
	Free-of-cost treatment facilities	Expand free ASM^a^ programs to district-level hospitals	Government regulation to ensure equitable access across regions
	Availability of healthcare providers	Strengthen 24/7 emergency neurology coverage	Staffing policies ensuring round-the-clock epilepsy care availability
Medication-Related Factors	Non-availability of ASMs	Establish a central procurement and supply chain monitoring	Law enforcement for continuous ASM^a^ availability at the hospital and retail pharmacies
	Medication side effects & pill burden	Conduct structured medication reviews to reduce pill burden	National guidelines recommending pharmacist-led medication review protocols
	Self-adjustment & perceived efficacy (dose skipping)	Routine counseling to check self-adjustment practices	National policy for mandatory adherence counseling at follow-up visits
	Perceived efficacy & tolerability of ASMs^a^	Monitor patient-reported outcomes during follow-up	Develop pharmacovigilance systems that capture adherence-related outcomes

^a^Antiseizure medications

### Limitations

Investigators collected data from PIMS, where persons with epilepsy mostly visit from low educational backgrounds. Due to illiteracy, the participants were not focused on the purpose of the study. Secondly, due to overcrowding, it became challenging to record the interviews. Third, when persons with epilepsy could not provide information, the interviewed CGs could not provide as rich data as persons with epilepsy could. Furthermore, due to the stigma, participants hesitated to share their information as the interviews were audio recorded. Rural or ethnic variations may not be fully represented, as most of the participants were from Islamabad, resulting in limited population diversity. However, the potential analyst bias was mitigated through team coding. Risk of meaning distortion during Urdu-English translation was minimized by repetitive team review and bilingual coders.

## Conclusions

This study shows that to address the challenges faced by persons with epilepsy and their CGs, the healthcare sector needs proactive strategies for integration. Findings on facilitators provide massive knowledge to healthcare providers to inculcate them in treatment strategies to provide efficient treatment services to persons with epilepsy facing poor treatment outcomes in LMICs. Our findings suggest the need for law enforcement in ASMs availability in hospitals, retail, community pharmacies, and pharmacist inclusivity in the multidisciplinary care process can overcome these barriers that have prevailed for years among persons with epilepsy.

## Supplementary Information

Supplementary Material 1.

Supplementary Material 2.

Supplementary Material 3.

## Data Availability

The datasets generated and/or analysed during the current study are not publicly available due to confidentiality and ethical considerations, but are available from the corresponding author on reasonable request.

## Abbreviations

ASMs: Antiseizure medications
CGs: Caregivers
COREQ: Consolidated Criteria for Reporting Qualitative Research
LMICs: Low- and middle-income countries
PIMS: Pakistan Institute of Medical Sciences
