# Supplementary material for: Barriers and facilitators of antiseizure medication adherence: a qualitative study among persons with epilepsy in Pakistan
Source: BMC Neurol. 2025 Oct 14;25:419. doi: 10.1186/s12883-025-04433-9 (PMC12522610; doi:10.1186/s12883-025-04433-9)
Supplement: Supplementary file 2 — Supplementary Material 2. [file 12883_2025_4433_MOESM2_ESM.docx]

**Interview Guide**

**Barriers and Facilitators of Antiseizure Drug Adherence: A Qualitative Study Among Persons with Epilepsy in Pakistan**

Name of interviewer..............................................................

Date of interview...................................................................

Interview code......................................................................

**Socio-Demographic Information**

1. Gender: Male Female
2. Age----------------------------------------

3. Education----------------------------------

4. Residence----------------------------------

5. Marital Status------------------------------

Q1. Could you please share with me what you know about your medicine for seizures?

**(Lifelong medicine treatment difficulties, risk of missing dose, benefit of medicine for seizures, importance of adherence, non-adherence consequences, health status)**

Q2. For how long have you been on treatment?

Q3. How did you take your medicines for seizures? Kindly share your medicine for seizures taking routine.

Q4. What did you do if you missed your daily medicine for seizures dose?

**(Probe how the persons with epilepsy takes the medicine for seizures dose, which was missed)**

Q5. Do you think religious belief will help you to get rid of this disease?

Q6. How did you manage to take your medicine for fits during Ramzan?

Q7. Kindly share why you stopped taking your medicines for seizures.

**(Start feeling better, tired of medicines, herbal/alternative treatment)**

Q8. Can you share why you started therapy & your experience concerning medicine for seizures?

**(Number of pills, color of pills, medicine for seizures stock out, size & smell of pills, dietary restrictions)**

Q9. What did you feel when you started taking your medicine for seizures?

**(Stress, sadness, happiness)**

Q10. Have you ever thought medicine for seizures did not work for you?

**(Self-assumption role)**

Q11. How do you feel about the services that you are receiving from PIMS Hospital?

**(Transportation cost, prescription charges, food cost, lab test cost, service/consultation fees)**

Q12. How did the hospital staff behave with you during treatment?

**(Healthcare setting support)**

Q13. Did your physician guide you about the side effects of medicine for seizures?

Q14. Did you share your health condition with anyone?

**(Medicines ingestion in front of relatives, friends, and the community)**

Q15. Kindly share about your family support.

Q16. Have you ever changed your medicine for seizures without talking to your doctor?

Q17. Were you satisfied with your treatment here?

Q18. What strategies have you found effective in remembering to take your medication particularly medicine for seizures?

Do you have any questions for me?

**Thank you very much for your time and good collaboration**
