# Supplementary material for: Barriers and facilitators of antiseizure medication adherence: a qualitative study among persons with epilepsy in Pakistan
Source: BMC Neurol. 2025 Oct 14;25:419. doi: 10.1186/s12883-025-04433-9 (PMC12522610; doi:10.1186/s12883-025-04433-9)
Supplement: Supplementary file 3 — Supplementary Material 3. [file 12883_2025_4433_MOESM3_ESM.docx]

**Supplementary File 2. Table 1. Additional or Supplementary Quotes- Barriers to Antiseizure Medication Adherence**

| **THEMES** | **SUBTHEMES** | **QUOTES** |
| --- | --- | --- |
| **PWE-related factors** | Disease-related stigma and discrimination | - My daughter changed schools because she feels ashamed when she takes her medicine for seizures in front of her friends. (CG 10) - I don’t like to sit with people as I prefer social isolation due to my condition. (P 03) - I usually prefer to share what I am going through with my family, but I do not share it with my friends or other people. (P 23) - When I go outside, I make a bag of medicines with cut tablets, one tablet from each strip. (P 27) |
|  |  |  |
|  | Psychological Issues | - If God has given me this disease, then give us money as well to cope with this disease, if not, then stop my breathing (P 05). - There is no cure for this disease; my family members are disturbed due to my condition. (P 09) - When I take my medicine for seizures, my head becomes sleepy. I cannot perform my daily duties, so my anxiety becomes severe when I'm around people. (P 24) - I am suffering from a lot of stress as no one listens to me about my condition. (P 07) |
|  | Financial instability | - It is difficult to manage transport costs, but we have no other option. We can only provide our son with treatment, but not quality treatment. (CG 21) - Gradually, we are becoming unable to bear the cost of treatment due to transportation costs and the increasing cost of medicines as my father must take six medicine for seizures in the morning and evening. (CG 16) |
|  | Comorbidities   - Dysphagia - Aspirational pneumonia | - He cannot take medicine for seizures because excessive saliva is coming from his mouth, and he is bedridden, so we can’t bring him to the hospital for a regular checkup. (CG 22) - He was on a liquid diet at home, but suddenly his fever and fits became uncontrolled. He has developed aspirational pneumonia and is on heavy antibiotics (CG 12) |
|  | Religious practices | - In Ramzan, I missed my medicine for seizure dose due to fasting (P 10) - For headaches, usually I opt for Dam Darood, but if it does not get better, I use my medicine for seizures (P 04) |
|  | Forgetfulness | - Usually, I forget to take my morning medicine for seizures dose due to my night shift at the hospital. (P 20) - She usually prefers not to go outside of the home; she only visits her grandmother's house and forgets to take her medicines there. (CG 06) - Sometimes due to anxiety, I forget to take my medicine for seizures because I feel suffocated. (P 20) |
| **Healthcare-related factors** | Short consultation times   - The doctor is occupied with too many patients, he can’t focus on me. As you can see this area is full of patients (P 11) - The doctor only visited in the morning and then the staff came to administer the IV medicines and gave us the tablets to give to our patient (CG 15) - We have only access to the counter, so we informed them we are not in direct contact with sir usually the staff comes and injects the medicines and informs us about the tablets on time (CG 23) - We have only 5-10 minutes to explain our daughter's condition and we are not satisfied after consultation (CG 18) - Medical tests performed in hospital were not correct and they did the wrong EEG, so we took our patient to Citi lab, and the reports were different there. (P 19). - For blood test reports, we must wait for two days because of this reason, he didn’t get timely treatment (CG 15) - The doctor only sees the previous prescription and writes new medicines, and my turn ends in five minutes (P 09) - I told my doctor that the Epival tablet causes stomach discomfort, so I’m not able to take it daily. He prescribed the same tablet on my new prescription. (P 26) - My doctor in my hometown Kashmir stopped my medicine for seizures after my first epileptic attack by wrongly diagnosing me with facial paralysis. (P 04) - We visited a hospital in our city Sialkot the doctor said our son is mentally ill. Take him to Lahore mental hospital. (CG 17) - Every time I visit this hospital, I get a new neurologist for my treatment. There is no specific doctor. I do not remember ever having been attended to by the same doctor (P 04) - I’m not sure who to trust as I have to repeat my story to doctors on every visit. (P 26) | |
|  | Neurology residents limited knowledge | - These doctors on training usually prescribe me the medicines after confirming them through mobile phones. (P 11) - On my previous follow-ups the doctors available here referred me to the psychiatric ward. (P 08) |
| **Medication-related factors** | Non-availability of ASMs | - If medicine for seizures are not available, then I purchase alternative medicines, but I do not prefer them. (P 09) - Sometimes, alternative medicines cause side effects, so I prefer to skip the medicine for seizures dose. (P 19) - In our village, original medicine for seizures are not available. If I take them, my condition does not get better. (P 19) - Usually, the medicine for seizures are short in Rawalpindi. If they are available, the prices are doubled. (P 27) - Our area has small medical stores they do not keep medicine for seizures. It has become a big problem for us. (CG 08) |
|  | Self-adjustment & perceived efficacy | - I prefer not to take tablets rather I go for IV injections. If the doctor prescribes it thrice daily, I reduce my dose to one time daily (P 14). - I took the dose of medicine for seizures in mid of the day and I had an epileptic attack 10 years back at that time doctor said to take half a tablet, but I gradually stopped the medicine and again suffered from an epileptic attack. (P 08) |
|  | Medication side effects & pill burden | - I usually skip my morning medicine for seizures dose and only take it at night, as it is easy for me. (P 19) - Due to his multiple medicines for pneumonia, we usually skip his some medicines doses. He cannot take all the medicines. (CG 24) - I’m on ten different medications on every visit they add more pills. I wonder if all these medicines are necessary for me. (P 21) |

**Supplementary File 2. Table 2. Additional or Supplementary Quotes -Facilitators to Antiseizure Medication Adherence**

| **THEMES** | **SUBTHEMES** | **QUOTES** |
| --- | --- | --- |
| **PWE-related factors** | Fear of epileptic attack pain | - I never miss my medicine for seizures dose because I'm afraid of dreadful seizures. (P 07) - Once my doctor told me that the medicine for seizures will protect you from epileptic episodes and pain after it. I follow his instructions religiously and feel better (P 25) |
|  | Family support | - Yes, she becomes aggressive sometimes for having multiple medicines to eat but I remind her that medicines are very important for you. (CG 09) - Yes, I have my family back, so I have survived despite this horrible disease. My sister used to look at me and she gave me medicine properly with full attention now I’m recovering. (P 12) - Alhamdulillah I have my family members. My husband also comes along with me for follow-ups and reminds me about medicine for seizures. (P 04) |
|  | Sense of responsibility | - I want to live a long life to care for my children, and I hope to see them as they grow old. (P 22) - I’m the only breadwinner in my family. My father is above 70 years old, and he can’t work. I visit the hospital regularly to stay epilepsy free for my family. (P 06) |
| **Healthcare-related factors** | Availability of healthcare providers | - Whenever I came to the hospital during an emergency condition, doctors were available, and they tackled my miserable condition immediately due to seizures. (P 07) - My son suffers from fever even in mild fever conditions, and when we take him to the hospital for treatment, neurologists are always available. (CG 11) - My doctor understands my condition. I can only share my condition with him. (P 20) |
|  | Free-of-cost treatment facilities | - This hospital is equipped with facilities to treat epilepsy symptoms other hospital refuses to treat my daughter. (CG 11) - My son was referred to this hospital because they have the only facilities and medication to treat epilepsy without any charges. (CG 19) - The neurology department has a crash cart trolley, they brought my son into the room and administered injections to control his uncontrolled seizures, which I did not see in other hospitals. (CG 05) - Every month I come to the hospital for to get free medicine for seizures. I have made a BAIT-UL-MAL form, so the doctor writes the medicines and every month gets the free medicine for seizures. (P 03) |
| **Medication-related factors** | Perceived treatment efficacy & tolerability | - Ten years back my doctor prescribed me medicine for my disease, due to which I am free from this disease and do not feel burdened. (P 14) - I’m using medicine for seizures since long time and now I didn’t remember when I suffered from epileptic episodes (P 22) - I become uncomfortable when I don’t take medicine for seizures. This medicine provides me with ease. (P 20) |
